# Identification of genomic regions of wheat associated with grain Fe and Zn content under drought and heat stress using genome-wide association study

**DOI:** 10.3389/fgene.2022.1034947

**Published:** 2022-10-21

**Authors:** Narayana Bhat Devate, Hari Krishna, V. P. Sunilkumar, Karthik Kumar Manjunath, C. N. Mishra, Neelu Jain, G. P. Singh, P. K. Singh

**Affiliations:** ^1^ Division of Genetics, ICAR-Indian Agricultural Research Institute, New Delhi, India; ^2^ ICAR- Indian Institute of Wheat and Barley Research, Karnal, India

**Keywords:** wheat, grain iron and zinc content, GWAS, drought stress, heat stress

## Abstract

Wheat is the staple food crop of global importance for its grain nutrient quality. Grain iron and zinc content of the wheat grain is an important quantitatively inherited trait that is influenced by the environmental factors such as drought and heat stress. Phenotypic evaluation of 295 advanced breeding lines from the wheat stress breeding program of IARI was carried out under timely sown irrigated (IR), restricted irrigated, and late-sown conditions at New Delhi during the cropping season of 2020–21, and grain iron (GFeC) and zinc (GZnC) contents were estimated from both control and treatments. A statistically significant increase in GFeC and GZnC was observed under stress conditions compared to that of the control. Genotyping was carried out with the SNPs from the 35K Axiom Breeder’s array, and marker–trait association was identified by GWAS analysis. Of the 23 MTAs identified, seven were linked with GFeC and sixteen were linked with GZnC. *In silico* analysis revealed a few important transcripts involved in various plant metabolism, growth, and development activities such as *auxin response factor*, *root UVB sensitive proteins*, *potassium transporter*, *glycosyl transferase*, *COBRA*, and *F-box-like domain.* The identified MTAs can be used for molecular breeding after validation and also for rapid development of micronutrient-rich varieties of wheat to mitigate hidden hunger.

## Introduction

Micronutrient deficiency, commonly known as “hidden hunger,” is mostly brought on by diets that are frequently dominated by staple foods that are poor in minerals and vitamins (Liu et al., 2019). Two billion people are affected by it globally, and it accounts for nearly 45% of all yearly fatalities in children under the age of 5. A higher risk factor for human health is the mineral deficiency of iron (Fe) and zinc (Zn), which affects around one-third of the population in underdeveloped nations (Rathan et al., 2022). In areas that are severely afflicted by micronutrient deficiencies, cereals make up the majority of daily dietary intake (Bouis et al., 2011). The micronutrient content of common cereals like wheat and rice, notably Fe and Zn, is below ideal levels.

According to Listman and Ordóez (2019), wheat is consumed by 2.5 billion people globally and is a staple food for 30% of the population, especially in developing nations (Lobell et al., 2011). Food fortification, supplementation, and diet diversity can all help solve the issue of micronutrient deficiency; however, these solutions are not long-lasting and are costly affairs, especially for rural poor people (Pfeiffer and McClafferty, 2007). Thus, increasing the nutritional content of crops by traditional and molecular methods, known as “biofortification,” has been accepted as an effective and sustainable method to address the issues related to micronutrient deficiency (Krishnappa et al., 2022).

The majority of the wheat-growing region is impacted by scattered showers and regular rising heat stress followed by heat waves. Lower grain filling time, reduced starch accumulation, and smaller seeds are all effects of heat and drought stress. Apart from this, the grain sink capacity decreased the most under heat and drought stress (Zahra et al., 2021). Warmer temperatures brought on by climate change and decreased water availability in the majority of spring wheat production regions will impact food and nutritional security (Velu et al., 2016). Accumulation of mineral nutrients in the grain is a complex process, including several genes, and is greatly impacted by environmental factors. A thorough understanding of the genetic regulation of nutritional characteristics and their relationship with grain yield is required to breed stable grain nutrient-containing varieties under drought and heat stress (Samineni et al., 2022).

In order to breed cereals like wheat for biofortification using MAS, it is important to have information about the genomic regions that control grain Zn and Fe concentration. Numerous QTLs and genes influencing the amount of these micronutrients have been identified as a result of genetic studies in wheat (Srinivasa et al., 2014; Tiwari et al., 2016; Krishnappa et al., 2017; Gupta et al., 2021). Genome-wide association studies (GWAS) are now the most widely used method for determining the genetic basis of complex characteristics such as grain Fe (GFeC) and Zn (GZnC) content. The GWAS has the advantages of increased QTL resolution, allele coverage, and the capacity to employ huge collections of readily available materials such as natural populations or advanced breeding lines. The GWAS has been used to show the genomic regions of grain iron and zinc content in many studies (Alomari et al., 2018; Bhatta et al., 2018; Velu et al., 2018; El-Soda and Aljabri, 2022; Krishnappa et al., 2022; Rathan et al., 2022). However, grain Zn and Fe content under drought and heat stress is not being explored much. Genomic regions governing complex traits are mostly adaptive QTLs that were detected under specific conditions of the environment such as drought stress (Khaled et al., 2022). Hence, our objective is to dissect the genomic regions related to grain Fe and Zn content under drought and heat stress conditions using GWAS in bread wheat (*Triticum aestivum*) advanced breeding lines.

## Materials and methods

### Plant material and field layout

The GWAS mapping panel consists of 295 advanced breeding lines developed from popular Indian and exotic varieties as described in Devate et al. (2022). All the materials were evaluated at the IARI, New Delhi (Indian Agricultural Research Institute, research farm located at 28°38′30.5″N, 77°09′58.2″E, 228 m AMSL) with three conditions, viz., timely sown irrigated (IR), timely sown drought (RI), and late-sown condition (LS). A total of six irrigations were given for the irrigated and late-sown trials, whereas one irrigation before and one irrigation after the sowing were provided for restricted irrigated trials. IR and RI trials were carried out in the first fortnight of November, whereas LS trials were carried out in the second fortnight of December to expose plants to the natural heat in later growing periods. The experiment was conducted in an augmented RCBD design with four checks and six blocks with a plot size of 1 m^2^ each.

### Phenotyping

Twenty random spikes from each line were collected in clean polyethylene bags and hand-threshed. Grain Fe and Zn contents (in mg/Kg) were estimated with 20-g seeds of each line through a high-throughput energy-dispersive X-ray fluorescence (ED-XRF) machine (model X-Supreme 8000; Oxford Instruments plc, Abingdon, United Kingdom) calibrated with glass bead-based values (Paltridge et al., 2012). Thousand kernel weight was measured by manual counting. The GWAS panel under all three conditions was phenotyped for GFeC, GZnC, and TGW. Phenotypic data were analyzed using the R package “augmentedRCBD” (Aravind et al., 2021) for ANOVA and adjusted means for each genotype under study. The z-test was used to compare the statistically significant differences between control and treatment means.

### Genotyping, population structure, and LD analysis

DNA isolation from leaf samples was carried out with the CTAB extraction method (Murray and Thompson 1980), followed by a DNA quality check through 0.8 percent agarose gel electrophoresis. Out of 295 genotypes, 282 DNA samples passed DNA quality thresholds and were genotyped using the Axiom Wheat Breeder’s Genotyping Array (Affymetrix, Santa Clara, CA, United States) with 35,143 SNPs. The SNP filtering was carried out for minor allele frequency (MAF) of <5%, missing data of >20%, and heterozygote frequency of >25% before further analysis. The remaining 10,546 SNPs with phenotypic data from 282 genotypes were used for further analysis. Population structure based on STRUCTURE software and molecular marker-based PCA analysis was conducted. Also, pairwise r^2^ values between markers and Linkage Disequilibrium decay plots were drawn as described in Devate et al. (2022).

### Analysis of data

Filtered 10,546 SNPs and adjusted means of GFeC and GZnC from each condition were used for genome-wide association analysis and were conducted using GAPIT v3 in R with the “BLINK” (Bayesian-information and Linkage-disequilibrium Iteratively Nested Keyway) (Huang et al., 2019) with PCA-based population structure as a fixed effect. Association model fitting was found using a Q–Q plot drawn with expected vs. observed *−log_10_(p)* value. Marker–trait associations (MTAs) in all three conditions for GFeC and GZnC were found with a significant *p* value cut-off at 0.001. Chromosomal maps with identified MTAs at respective positions in Million Base-pairs (mb) on respective chromosomes with the name of associated traits were drawn with MapChart v2.32 (Voorrips, 2002).

Genotypes were grouped into two classes for each allele of MTAs, and their means were compared to identify trait-increasing and trait-decreasing alleles. Sequence information of significant markers was used for similarity search with the IWGSC reference genome with the basic local alignment search tool (BLAST) using the Ensembl Plants database (http://plants.ensembl.org/index.html) of the bread wheat genome [IWGSC (RefSeq v1.0)]. The gene coding regions located within and near the 100-kb flanking region of the MTAs were listed with transcript ID and described the protein (if any).

## Results and discussion

**FIGURE 1:**
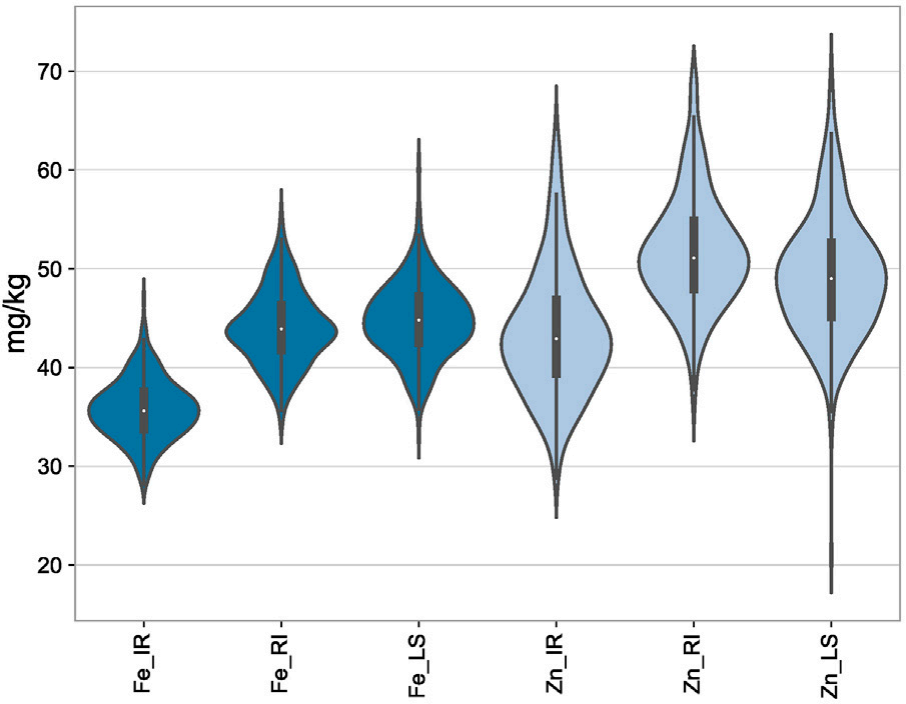
Depiction of the distribution of GFeC and GZnC under IR, RI, and LS conditions through violin plots.

Analysis of variance in all the three conditions, viz., IR, RI, and LS, showed significant variation among the genotypes for GFeC, GZnC, and TGW. The mean values of GFeC and GZnC were increased under LS and RI conditions compared to those under IR (Figure 1), which was significant at both the one-tailed and two-tailed z tests at a *p*-value of 0.05. The average grain iron content under irrigated conditions was 35.7 mg/kg and was increased to 44.03 and 44.7 mg/kg under RI and LS conditions, respectively. Similarly, the average grain zinc content was increased from 43.52 to 51.86 mg/kg under RI and 49.29 mg/kg under LS conditions (Table 1). The decrease in TGW can be attributed to decreased assimilation of photosynthates and reduced grain sink capacity under drought and heat stress, ultimately leading to small and shriveled grains and reduced starch content in the endosperm (Zahra et al., 2021). It was observed that increased GFeC and GZnC under drought may be due to the shrinkage effect of grains, whereas under non-stressed conditions, due to high yield, the dilution effect of GFeC and GZnC results in a lower concentration. However, the nutrient yield per unit area decreases under stress (Velu et al., 2016).

**TABLE 1:** Summary and correlation of GFeC, GZnC, and TGW under IR, RI, and LS treatments in the GWAS panel evaluated at Delhi 2020–21.

Treatment	Trait	Mean ± SD	Range	CV	Hbs	Percent increase/decrease	Z-test	Correlation		
						IR with RI and LS	IR with RI and LS	GFeC	GZnC	TGW
IR	GFeC	35.7 ± 3.04	28.35–47.35	5.56	58.59	—	—	1	0.2***	−0.09
	GZnC	43.52 ± 6.85	28.25–65.67	5.67	86.95	—	—		1	−0.04
	TGW	38.82 ± 4.48	24.92–57.29	6.2	75.72	—	—			1
RI	GFeC	44.03 ± 3.59	34.88–54.57	4.12	77.91	23.33%	89.75**	1	0.41***	−0.17**
	GZnC	51.86 ± 6.1	35.83–70.44	5.02	81.66	19.16%	39.83**		1	−0.21***
	TGW	32.98 ± 4.83	21.73–51.6	6.28	82.26	−15.04%	0.02^NS^			1
LS	GFeC	44.7 ± 4.01	32.8–59.85	4.19	78.08	25.21%	85.41**	1	0.36***	0.05
	GZnC	49.29 ± 6.51	21.85–71.71	4.73	87.14	13.25%	26.89**		1	−0.16**
	TGW	36.28 ± 4.03	23.63–48.62	5.82	73.53	−06.54%	0.03^NS^			1

*p < 0.05, **p < 0.01, and ***p < 0.001.

A correlation study among the studied traits, viz., GFeC, GZnC, and TGW, showed a significant negative correlation between TGW and grain Fe and Zn content under RI conditions (at the *p* values of 0.001 and 0.01, respectively) and grain Zn condition under LS conditions (at a *p*-value of 0.01), whereas no significant correlation was observed between TGW and grain Fe and Zn under control. GFeC and GZnC had a significant positive correlation among themselves under all the three treatment conditions, with a cut-off *p*-value of 0.001 (Table 1). Having a positive correlation between GFeC and GZnC can be efficiently utilized during a breeding program to achieve simultaneous improvement of both traits (Rathan et al., 2022). Broad sense heritability of GFeC, GZnC, and TGW was medium to high under all the three treatments, indicating the predominance of additive gene action. Traits governed by additive gene action and having positive correlation can be improved together efficiently in spite of environmental influences (Borah et al., 2018).

The GWAS panel under study had two subpopulations based on STRUCTURE and PCA scatter plots. Linkage-disequilibrium (LD) decay block size was shown to be 5.24, 5.26, and 9.22 MB for the A, B, and D genomes, respectively, and a block of 7.15 MB was observed for the whole genome (Devate et al., 2022). The effect of the population structure of the association panel on the GWAS analysis is addressed by taking them as a covariate to ensure true association. LD decay is used to determine the number of markers required to be used in the GWAS. The rate of LD decay varies depending on the rate of recombination between the marker pairs (Borah et al., 2018). In general, in outcrossing crop species such as maize, LD decays rapidly, in contrast to self-pollinated crops, which show slow decay, as in wheat (Yu et al., 2014; Roncallo et al., 2021).

**FIGURE 2:**
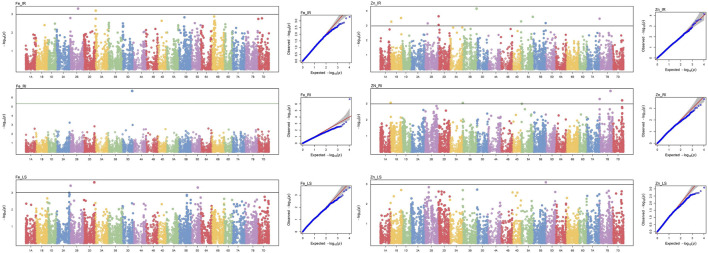
Manhattan and respective Q–Q plots of significant associations for GFeC and GZnC under IR, RI, and LS conditions.

**FIGURE 3:**
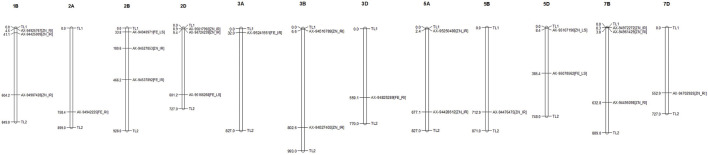
Distribution and position (in Mb) of identified MTAs at their respective chromosomes with associated traits.

Genome-wide 10,546 SNPs over 282 genotypes were used to identify the markers associated with grain Fe and Zn contents across control (IR), drought (RI), and heat stress (LS) treatments. Of the 23 MTAs identified, seven MTAs were detected for GFeC, and 16 for GZnC (Table 2) can be visualized by a Manhattan plot with the threshold at *p < 0.001* (Figure 2) and their position on respective chromosomes in Figure 3. Trait-increasing allele and trait-decreasing allele for each associated marker are given in Table 2. The identified MTAs were treatment-specific, implicating differential expression of genes under different stress conditions. The putative candidate genes located within the 100-kb region of the linked MTAs were identified by a BLAST search against the IWGSC reference genome at the Ensembl Plants database.

**TABLE 2:** Significant marker–trait associations for GFeC and GZnC along with the details of trait-increasing and trait-decreasing alleles.

Trait	SNP	Chromosome	Position (MB)	*p*-value	−*log10(p)*	Decreasing allele	Increasing allele
Fe_IR	AX-94537892	chr2B	466.21	0.000506	3.295525	A	G
Fe_IR	AX-95241551	chr3A	31.95	0.000651	3.186231	C	T
Fe_LS	AX-95166268	chr2D	601.18	0.000241	3.617943	T	C
Fe_LS	AX-94940971	chr2B	33.84	0.000383	3.417198	C	T
Fe_LS	AX-95078562	chr5D	385.39	0.000491	3.309289	C	T
Fe_RI	AX-94825289	chr3D	559.08	1.90E-07	6.720941	A	T
Fe_RI	AX-94942225	chr2A	758.39	0.000587	3.231101	C	T
Zn_IR	AX-94527403	chr3B	802.60	7.08E-05	4.149945	C	A
Zn_IR	AX-95017992	chr2D	8.93	0.000236	3.627449	A	C
Zn_IR	AX-94428612	chr5A	677.13	0.000255	3.592764	G	A
Zn_IR	AX-94987428	chr1B	604.15	0.000308	3.510902	C	T
Zn_IR	AX-94972272	chr7B	0.27	0.000342	3.466551	A	G
Zn_IR	AX-95250489	chr5A	2.41	0.000528	3.276998	A	G
Zn_IR	AX-94425009	chr1B	41.09	0.000548	3.26091	A	G
Zn_IR	AX-94476475	chr5B	712.86	0.000685	3.164529	T	C
Zn_IR	AX-94527853	chr2B	180.56	0.000721	3.14236	G	A
Zn_IR	AX-94724239	chr2D	9.35	0.000902	3.044792	G	A
Zn_LS	AX-95107190	chr5D	8.35	0.000812	3.090692	A	C
Zn_RI	AX-94495098	chr7B	632.80	0.000164	3.785761	A	G
Zn_RI	AX-94961429	chr7B	3.80	0.000518	3.285462	T	A
Zn_RI	AX-94702925	chr7D	552.93	0.000625	3.203826	C	T
Zn_RI	AX-94925767	chr1B	4.46	0.000893	3.048914	G	C
Zn_RI	AX-94516789	chr3B	6.56	0.000901	3.045095	C	T

The MTAs identified for grain Fe content under IR were located on chromosomes 2B and 3A at the positions of 466.2 and 31.9 mb, respectively, in concordance with those previously noted by Rathan et al. (2022) on chromosomes 2B and 3A and on 3A by Bhatta et al. (2018). A putative candidate gene (*TraesCS2B02G326400*) near marker AX-94537892 codes for the “*auxin response factor*,” which has a role in various growth and development and response to abiotic stresses, such as drought, salt, or cold (Xu et al., 2020). The same region contains another protein-coding region (*TraesCS2B02G326300*) for “*cleft lip and palate transmembrane 1*,” which is less studied in plants. Putative genes near the other SNP marker AX-95241551 were the “*WD40/YVTN repeat-like-containing domain superfamily*” and “*root UVB sensitive family*,” the roles of which in supramolecular interactions (Guerriero et al., 2015) and sunlight-dependent root development (Tong et al., 2008) were confirmed by previous studies, respectively. As protein interaction and root architecture are important criteria for nutrient uptake, they might be linked with the grain Fe content. Similarly, under drought stress conditions, AX-94825289 and AX-94942225 were identified on chromosomes 3D and 2A, respectively, and are linked with GFeC, present in the region having putative candidate genes for “*potassium transporter*” and “*ubiquinone biosynthesis protein.*” Potassium transporter has a role in concentration gradients of protons, homeostasis of monovalent cations (Gierth and Mäser, 2007), and symporter and antiporter transport of the ions, whereas ubiquinone protects the membrane from free radical-induced oxidative damage (Pobezhimova and Voinikov, 2000), which is commonly observed under stress. Three unique MTAs were identified under LS conditions for grain iron content on chromosomes 2D, 2B, and 5D. They were putatively linked to transcripts having a role in protein modification (*peptidyl-tRNA hydrolase* and *peptidyl-prolyl cis-trans isomerase*), carbohydrate binding (*bulb-type lectin domain*), and enzymes involved in lipid signaling pathways (*phospholipase C/P1 nuclease domain*) (Table 3).

**TABLE 3:** Putative candidate genes in the 100-kb region of the linked marker with the protein produced from them.

Trait	SNP	Position	Transcript ID	Protein
FE_IR	AX-94537892	2B: 474382601..474388204 (−strand)	TraesCS2B02G326400	➢ Auxin response factor
		2B: 474355042..474361352 (−strand)	TraesCS2B02G326300	➢ Cleft lip and palate transmembrane 1
FE_IR	AX-95241551	3A: 32643085..32647110 (+strand)	TraesCS3A02G054700	➢ WD40/YVTN repeat-like-containing domain superfamily
		3A: 32647241..32651665 (−strand)	TraesCS3A02G054800	➢ Root UVB sensitive family
		3A: 32621252..32625702 (+strand)	TraesCS3A02G054600	➢ P-loop containing nucleoside triphosphate hydrolase
FE_LS	AX-95166268	2D: 604123946..604130138 (+strand)	TraesCS2D02G507500	➢ Staphylococcal nuclease (SNase-like), OB-fold
		2D: 604146523..604150893 (+strand)	TraesCS2D02G507600	➢ Peptidyl-tRNA hydrolase, PTH2
		2D: 604151044..604155857 (−strand)	TraesCS2D02G507700	➢ Phospholipase C/P1 nuclease domain superfamily
FE_LS	AX-94940971	2B: 38339312..38342120 (−strand)	TraesCS2B02G067000	➢ Bulb-type lectin domain superfamily
		2B: 38348364..38351197 (+strand)	TraesCS2B02G067100	➢ FKBP-type peptidyl-prolyl cis-trans isomerase domain
FE_LS	AX-95078562	5D: 388220089..388223638 (+strand)	TraesCS5D02G284600	➢ Gnk2-homologous domain
		5D: 388268407..388270613 (−strand)	TraesCS5D02G284700	➢ Tetratricopeptide-like helical domain superfamily
FE_RI	AX-94825289	3D: 560443926..560449941 (+strand)	TraesCS3D02G450800	➢ Potassium transporter
		3D: 560449825..560456397 (−strand)	TraesCS3D02G450900	➢ Spermatogenesis-associated protein 20
FE_RI	AX-94942225	2A: 762552292..762554307 (−strand)	TraesCS2A02G551800	➢ Tetratricopeptide-like helical domain superfamily
		2A: 762554483..762556597 (+strand)	TraesCS2A02G551900	➢ Ubiquinone biosynthesis protein Coq4
		2A: 762556814..762564083 (−strand)	TraesCS2A02G552000	➢ Clathrin, heavy chain/VPS, 7-fold repeat
		2A: 762577383..762581944 (+strand)	TraesCS2A02G552100	➢ Tetratricopeptide-like helical domain superfamily
		2A: 762528939..762530243 (−strand)	TraesCS2A02G551700	➢ F-box associated interaction domain
ZN_IR	AX-94527403	3B: 820348909..820353167 (−strand)	TraesCS3B02G570600	➢ Protein kinase-like domain superfamily
		3B: 820363175..820364327 (−strand)	TraesCS3B02G570500	➢ Myb/SANT-like domain
ZN_IR	AX-95017992	2A: 9870676..9873085 (−strand)	TraesCS2A02G017600	➢ Domain of unknown function DUF1618
		2A: 9864423..9870187 (+strand)	TraesCS2A02G017500	➢ 2-Oxoacid dehydrogenase acyltransferase, catalytic domain
		2A: 9838071..9840946 (+strand)	TraesCS2A02G017400	➢ FACT complex subunit Spt16 N-terminal lobe domain
		2A: 9831458..9833452 (−strand)	TraesCS2A02G017300	➢ FBD domain
ZN_IR	AX-94428612	5A: 678935942..678937957 (−strand)	TraesCS5A02G513300	➢ Ribosomal protein L21e
		5A: 678941500..678946425 (−strand)	TraesCS5A02G513400	➢ Methyltransferase type 12
		5A: 678965411..678966822 (+strand)	TraesCS5A02G513500	➢ Ribosomal protein L5 domain superfamily
		5A: 678966202..678969731 (−strand)	TraesCS5A02G513600	➢ Pectin lyase fold/virulence factor
ZN_IR	AX-94987428	1B: 611299086..611301010 (−strand)	TraesCS1B02G373600	➢ 6-Phosphogluconate dehydrogenase, decarboxylating
ZN_IR	AX-94972272	7B: 265308..267721 (−strand)	TraesCS7B02G001200	➢ F-box-like domain superfamily
		7B: 261777..263347 (+strand)	TraesCS7B02G001100	➢ NTF2-like domain superfamily
ZN_IR	AX-95250489	5A: 3318157..3321929 (−strand)	TraesCS5A02G003200	➢ Glycosyl transferase, family 14
		5A: 3315802..3317881 (−strand)	TraesCS5A02G003100	➢ Longin-like domain superfamily
		5A: 3312509..3313435 (+strand)	TraesCS5A02G003000	➢ Chaperone J-domain superfamily
		5A: 3306994..3308561 (−strand)	TraesCS5A02G002900	➢ Chalcone/stilbene synthase, N-terminal
ZN_IR	AX-94425009	1B: 45453621..45459411 (−strand)	TraesCS1B02G058700	➢ Beta-hexosaminidase, eukaryotic type, N-terminal
		1B: 45447127..45450887 (−strand)	TraesCS1B02G058600	➢ Protein-tyrosine phosphatase-like
ZN_IR	AX-94476475	5B: 714519498..714521198 (−strand)	TraesCS5B02G571900	➢ NTF2-like domain superfamily
		5B: 714565705..714567173 (−strand)	TraesCS5B02G572400	➢ Glutathione S-transferase, N-terminal
ZN_IR	AX-94527853	2B: 188627646..188629785 (+strand)	TraesCS2B02G201400	➢ COBRA and plant
		2B: 188674693..188676234 (+strand)	TraesCS2B02G201600	➢ WD40-repeat-containing domain superfamily
ZN_IR	AX-94724239	2D: 9539575..9541245 (+strand)	TraesCS2D02G019400	➢ P-loop containing nucleoside triphosphate hydrolase
		2D: 9560490..9570556 (+strand)	TraesCS2D02G019600	➢ Protein kinase-like domain superfamily
ZN_LS	AX-95107190	5D: 8234067..8262668 (+strand)	TraesCS5D02G014500	➢ Ankyrin repeat-containing domain superfamily
ZN_RI	AX-94495098	7B: 632793597..632795681 (−strand)	TraesCS7B02G368500	➢ Pentatricopeptide repeat
		7B: 632799627..632800394 (−strand)	TraesCS7B02G368600	➢ TFIIS/LEDGF domain superfamily
ZN_RI	AX-94961429	7B: 3789679..3794857 (+strand)	TraesCS7B02G006700	➢ Ubiquinone biosynthesis O-methyltransferase
		7B: 3795056..3797564 (−strand)	TraesCS7B02G006800	➢ Transcription elongation factor 1
ZN_RI	AX-94702925	7D: 552946865..552951091 (+strand)	TraesCS7D02G433000	➢ Putative S-adenosyl-L-methionine-dependent methyltransferase
		7D: 552961791..552966500 (+strand)	TraesCS7D02G433100	➢ GDP-fucose protein O-fucosyltransferase
ZN_RI	AX-94925767	1B: 4475908..4479190 (+strand)	TraesCS1B02G008200	➢ Snf7 family
		1B: 4480185..4481605 (−strand)	TraesCS1B02G008300	➢ Protein kinase-like domain superfamily
ZN_RI	AX-94516789	3B: 6554790..6558914 (+strand)	TraesCS3B02G016000	➢ Fatty acyl-coenzyme A reductase, NAD-binding domain
		3B: 6533011..6534111 (+strand)	TraesCS3B02G015800	➢Phosphatidylethanolamine-binding protein

GZnC under irrigated conditions was linked with 10 different SNPs which were located on 1B, 2B, 2D, 3B, 5A, 5B, and 7B. Previous studies reported stably expressing MTAs on 5B (Cu et al., 2020), 7B (Rathan et al., 2022), 3B, and 5A (Alomari et al., 2018). Genomic regions of GZnC-linked SNPs of the current study contained important genes coding for *glycosyl transferase (TraesCS5A02G003200)*, *COBRA*, *plant (TraesCS2B02G201400)*, *WD40-repeat-containing domain superfamily (TraesCS2B02G201600)*, and *P-loop containing nucleoside triphosphate hydrolase (TraesCS2D02G019400*), whose roles in flavonoid biosynthesis (Yao et al., 2019), orientation, and cell expansion in Arabidopsis root (Roudier et al., 2002), supramolecular interactions (Guerriero et al., 2015), and metallochaperones and metalloenzymes (Vaccaro and Drennan, 2022) were reported in previous studies. Flavonoids play a crucial role in the growth and development of plants, which might have an influence on the quality of the product and its nutrient status. Similarly, nucleoside triphosphate hydrolase plays a crucial role as a metallochaperone, which might have a direct or indirect role in the GZnC of wheat. Apart from these, *protein kinase*, *Myb/SANT-like domain*, *FACT complex*, *FBD domain*, *ribosomal protein L21e*, *methyltransferase type 12*, *ribosomal protein L5 domain*, and *F-box-like domain* are other important genes present in the candidate regions (Table 3).

Grain Zn content under drought stress was linked with SNP markers located on 1B, 3B, 7B, and 7D. A putative candidate gene near the linked SNP AX-94495098 was *TraesCS7B02G368500*, which codes for “*pentatricopeptide repeat*,” which controls post-transcriptional regulation of many genes at the RNA level (Zhang et al., 2020). Other two MTAs, AX-94961429 and AX-94702925, were linked to the different genes that code for *methyltransferase*, which has a crucial role in biochemical reaction and plant metabolism (Moffatt and Weretilnyk, 2001). The *ubiquinone biosynthesis* gene was found to be linked in RI condition with GZnC like GFeC as mentioned earlier and has a crucial role in managing free radical-induced oxidative damage. Marker AX-94516789 is linked with the gene coding for “*phosphatidylethanolamine-binding protein*” and has a role in the morphological switch between shoot and flower structure and signal transduction (Banfield and Brady, 2000).

Under heat stress, i.e., LS condition, only one marker (AX-95107190) on 5D showed association with GZnC. A candidate gene at the genomic region of SNP was found to be coded for an *ankyrin repeat-containing domain*, whose critical role in plant growth and development, hormone response, and response to biotic and abiotic stresses was discovered in previous studies (Lopez-Ortiz, et al., 2020). Stress tolerance and nutrient assimilation in the plant involve many complex pathways governed by many related and superfamily genes. Putative candidate genes identified here have a direct or indirect influence on various plant growth and development processes and may play a role in nutrient uptake and grain nutrient content.

## Conclusion

The GWAS panel used in this study with 282 advanced breeding lines of wheat has shown that GFeC, GZnC, and TGW were complex traits, inherited quantitatively, and their expression was highly influenced by abiotic stress factors such as drought and heat stress. A positive correlation between the GFeC and GZnC and their high heritability indicate that simultaneous improvement of both the traits can be possible. Out of 23 MTAs identified under IR, RI, and LS conditions, seven were linked to GFeC and sixteen were linked to GZnC. The identified MTAs were located near novel candidate genes and have a direct or indirect effect on traits. Several identified putative candidate genes encode important molecular functions such as metallochaperones, root architecture orientation, ionic homeostasis, and abiotic stress response. Further validation of identified MTAs can be carried out and is useful in marker-assisted selection programs to develop biofortified varieties.

## Data Availability

The original contributions presented in the study are publicly available. This data can be found here: https://doi.org/10.5061/dryad.qnk98sfkw.
